# Frequency and risk factors for arm lymphedema after multimodal breast-conserving treatment of nodal positive breast Cancer – a long-term observation

**DOI:** 10.1186/s13014-019-1243-y

**Published:** 2019-03-07

**Authors:** Julia Rupp, Catarina Hadamitzky, Christoph Henkenberens, Hans Christiansen, Diana Steinmann, Frank Bruns

**Affiliations:** 1 Department of Radiation Oncology, Hannover Medical School (MHH), Carl-Neuberg-Straße 1, 30625 Hannover, Germany; 2 Department of Gynecology and Obstetrics, DIAKOVERE Hospital Henriettenstift, Hannover, Germany; 3 Department of Plastic, Aesthetic, Hand and Reconstructive Surgery, Hannover Medical School, Hannover, Germany; 4 Practice for Lympho-Vascular Diseases, Bahnhofstraße 12, Hannover, Germany

**Keywords:** Lymphedema, Breast cancer, BCRL, Radiotherapy, Breast-conserving surgery, Axillary surgery, Quality of life

## Abstract

**Background:**

Arm*-*lymphedema is a major complication after breast cancer. Recent studies demonstrate the validity of predicting Breast Cancer Related Lymphedema (BCRL) by self-reports. We aimed to investigate the rate of BCRL and its risk factors in the long-term using self-reported symptoms.

**Methods:**

Data was collected from 385 patients who underwent multimodal therapy for nodal positive breast cancer, including breast conserving surgery, axillary dissection, and local or locoregional radiotherapy. Two validated questionnaires were used for the survey of BCRL (i.e. LBCQ-D and SDBC-D). These were analysed collectively with retrospective data of our medical records.

**Results:**

23.5% (*n* = 43) suffered a permanent BCRL (stage II-III) after a median follow-up time of 10.1 years (4.9–15.9 years); further 11.5% (*n* = 23) reported at least one episode of reversible BCRL (Stage 0-I) during the follow-up time. 87.1% of the patients with lymphedema developed this condition in the first two years. Adjuvant chemotherapy was a significant risk factor for the appearance of BCRL (*p* = 0.001; 95%-CI 7.7–10.2).

**Conclusions:**

Breast cancer survivors face a high risk of BCRL, particularly if axillary dissection was carried out. Almost 90% of BCRL occurred during the first two years after radiotherapy. Self-report of symptoms seems to be a suitable instrument of early detection of BCRL.

## Background

Breast cancer is the most frequent malignant disease among women, with an estimated 1.67 million new cases diagnosed worldwide each year [1]. Modern treatment is multimodal and can include surgical resection (lumpectomy or mastectomy with sentinel node biopsy or axillary dissection), radiotherapy (with or without regional nodal irradiation), chemotherapy, anti-HER2-therapy, and/or endocrine therapy [2, 3]. This approach has resulted in documented survival benefits with 5-year overall survival rates of nearly 90% based on data from SEER 18 (2006–2013) [4]. Therefore, the effects of post-treatment-related complications on long-term quality of life have become increasingly important.

Breast-cancer related arm-lymphedema (BCRL) is one of the most severe side effects of breast cancer treatment [5] and is known to have a profoundly negative impact on the quality of life (QoL) [6–8]. Women treated for breast cancer face a lifetime risk of developing lymphedema [9], a chronic swelling of the arm and sometimes concomitantly of the breast /trunk. This is caused by an accumulation of protein-rich interstitial fluid, which leads to chronic inflammation with later fibrosis [10]. BCRL not only causes abnormal swelling but also a variety of lymphedema-associated symptoms. They primarily result from the obstruction or disruption of the lymphatic system due to the breast cancer treatment [11]. The limb volume increase seems to be directly proportional to the number of reported symptoms [9, 12]. Also, the presence of associated symptoms in the affected limb may indicate a latent stage of BCRL not yet detected by objective measures [11–13]. Armer et al [9] could demonstrate the validity of predicting BCRL by self-reported symptoms using a special questionnaire, i.e. the Lymphedema Breast Cancer Questionnaire (LBCQ).

Approximately one in five breast cancer survivors will develop BCRL [14]. But the frequency of BCRL after breast cancer treatment varies greatly in the literature ranging up to 56% in older publications [15]. This seems to be a result of methodological differences regarding the definition and measurement of BCRL through patients or physicians, length of follow up and types of breast cancer treatment [16]. Interestingly, some studies report an increased frequency of BCRL during follow-up time [15, 17].

To the present date, data on this subject including a follow up of considerably more than five years, as in our collective, is rare. The aim of this retrospective study was to assess the frequency of BCRL with a minimum follow-up time of five years. Additionally, we studied the effects of individual risk factors on the occurrence of BCRL when multimodal breast cancer therapy included complete axillary lymph node dissection (ALND), as this is one of the established risk factors for BCRL [13].

## Methods

Between 2000 and 2010, 385 consecutive patients with histologically proven nodal positive non-metastatic breast cancer were first treated with breast-conserving surgery and complete axillary lymph node dissection (ALND). Thereafter, a postoperative radiotherapy was performed in our department. All patients received a whole breast irradiation (WBI) using tangential fields in supine position with a median total dose of 50.4 Gy (range, 40.0 to 50.4 Gy). 75 patients received irradiation of supraclavicular lymph nodes (SCRT) with a median dose of 45 Gy. The superior, inferior, lateral, and medial borders of the field of SCRT were the upper border of the supraclavicular fossa, 1 cm above the match line of tangential beams of WBI, acromioclavicular joint, and 1 cm from the spinal cord; intentionally, axillary lymph nodes were not irradiated completely for the purpose of lymphedema prevention. Additionally, 65 patients received a boost irradiation to the primary tumor bed with a median dose of 10 Gy.

Information about tumor, patient and treatment characteristics, survival rate as well as presence of BCRL was collected retrospectively from our medical records and radiation therapy reports. The clinical TNM status was noted using the standardized tumor/node/metastasis (TNM) classification system (7th edition) [18]. The epidemiologic cancer registry was retrieved for updated information about cancer recurrence and life status. The median number of resected lymph nodes during ALND was 17.5 (range 2–55). In the few cases (*n* = 23; 11,5%), where neoadjuvant chemotherapy was necessary, we classified the pre-therapeutic clinical TNM status as ypTNM. Here, N+ was noted as at least N1, depending on the result of the ALND.

Two validated questionnaires were used in order to analyse self-reported symptoms and global quality of life. First, the validated questionnaire LBCQ (*University of Missouri Lymphedema and Breast cancer Questionnaire*), in a linguistic validated German translation (LBCQ-D) [19] was used to self-assess the rate of BCRL: Patients indicating arm swelling and/or arm heaviness and/or swelling including pitting were then considered to have BCRL. The second questionnaire named *structure demand for breast cancer* (SDBC-D) was developed and validated in German language in our department. It allowed screening of late side effects of radiotherapy in breast cancer patients [20]. This second questionnaire was only partially used with focus on selected questions concerning late radiation effects and BCRL detection. The global QoL at the date of survey was measured with an 11-point Numerical Rating Scale (NRS) analogue to the commonly used pain rating scale [21]. A higher score corresponded to a better QoL. As there was no previous data comparing the QoL of patients after nodal positive breast cancer therapy with the QoL of the general population, we used a direct comparison between patient groups, instead of artificially setting a cut off QoL value. If the subjective presence of lymphedema was inconsistent when comparing both questionnaires, then the LBCQ questionnaire was considered deciding. If information was imprecise, patients were additionally contacted via telephone for clarification. In cases where the questionnaire was not filled out correctly but the patient had used the free text areas to clearly point out the presence of BCRL, we classified it as having developed the condition. Two clinical experienced researchers analysed the presence and stage of BCRL based on the above-mentioned questionnaires, following a double-blinded dual control principle. Clinical examination was not performed. The investigation and survey was performed with the approval of the regional Hannover Medical School ethics committee.

The questionnaire analysis allowed partial correspondence of lymphedema severity to the clinical stages stated by the International Society of Lymphology [22]. If a singular episode of symptoms was indicated, it was classified as a completely reversible BCRL (Stage 0). If lymphedema was subjectively present twelve months prior to the study but not during follow up, patients were classified as having recurrent BCRL (stage I). Even though the distinction between clinical stages II and III was not possible, we assumed the worst case to prevent false negatives. Patients with symptoms of BCRL for more than 12 months were classified as chronically in Stage III.

BCRL outcome was measured from the date of the first radiation to the last date of follow-up. Patients alive and without evidence of disease were censored at the date of their last follow-up. If the annual specification only included month and year, we used the first day of the month for calculation. If the annual specification only included the year, we used the first of January for calculation. This concerns the information of the beginning of BCRL and the follow-up period (start of irradiation until month of participation in the survey).

### Statistics

The time-dependent BCRL rates were estimated with the Kaplan-Meier method using the log-rank test with a *p* = 0.05 significance level; *p*-values > 0.1 were considered as not significant. As we assumed that the assessed endpoint “frequency of lymphedema” was strongly time-dependent we used time to event analysis for calculation. Kaplan-Meier analysis and the log rank test were used for univariate analysis to identify significant risk factors.

All tested patient-, tumor-, and treatment-related parameters are listed in Table 1. All univariate significant parameters were used for a multivariate Cox Regression Analysis, using the backwards elimination to find the most important parameter. To analyse the correlation between QoL and the frequency of BCRL we used cross tables and a Chi-squared test. To analyse the coincidence between lymphedema-associated symptoms and the occurrence of BCRL we used cross tabulation. All analysis were performed using the statistical software package SPSS, version 24 (open source).

**Table 1:** Patients’ characteristics and treatment modalities

Variable		
Age	*Median (range)*	57.0 (34.3–79.9)
Body mass index (BMI)	*Median (range) t0* ^*a*^	25.0 (18.7–63.5)
	*Median (range) t1* ^*a*^	25.5 (16.4–65.3)
pT stage		*Number of patients (%)*
	I	97 (48.5%)
	II	95 (47.5%)
	III	4 (2.0%)
	IV	3 (1.5%)
pN stage		
	1	165 (82.5%)
	2	28 (14.0%)
	3	7 (3.5%)
Grading		
	1	13 (6.5%)
	2	144 (57.0%)
	3	69 (34.5%)
Tumor size (mm)	*Median (range)*	19 (0–90)
Resection volume (g)	*Median (range)*	50 (5–931)
Resected lymph nodes (n)	*Median (range)*	17.5 (2–55)
Involved lymph nodes (n)	*Median (range)*	2 (0–22)
Hormone therapy		*Number of patients (%)*
	*Yes*	152 (76.0%)
	*No*	43 (21.5%)
Chemotherapy	Total	
	*yes*	156 (78.0%)
	*no*	44 (22.0%)
	Adjuvant	
	*Yes*	133 (66.5%)
	*No*	67 (33.5%)
	Neoadjuvant	
	*Yes*	23 (11.5%)
	*No*	177 (88.5%)
Radiation volume	Breast	
	*yes*	200 (100%)
	SCRT	
	*Yes*	75 (37.5%)
	*No*	125 (62.5%)
	Boost (breast)	
	*Yes*	65 (32.5%)
	*No*	135 (67.5%)
Follow up (years)	*Median (range)*	10.1 (4.9–15.9)

^a^*t0* = at time of irradiation; *t1* = at time of survey

## Results

At the time of survey, 88 of 385 patients had died (22.9%); in most cases we had no information about the cause of death. Eleven patients were excluded from this survey, as one year after having breast-conserving surgery a secondary mastectomy had been necessary. Two further patients were excluded because only sentinel lymph node biopsy (SLNB) was performed, ALDN being one of the inclusion criteria. 284 Patients (73.8%) remained in the survey and were addressed via post including the questionnaires. A total of 200 patients (70.4%) answered the questionnaire and entered the detailed analysis. A summary of the patient’s baseline characteristics is listed in Table 1.

### Incidence of lymphedema

Median follow-up time was 10.1 years. 70 Patients (35%) showed symptoms of BCRL irrespective of duration and severity. Further 13 Patients (6.5%) showed symptoms of breast and/or axillary edema, but no arm edema. Of all BCRL patients, eight patients (4%) indicated a completely reversible BCRL (Stage 0) within the first year after radiotherapy. 15 patients (7.5%) had a reversible but recurrent LE (Stage I) and 47 patients (23.5%) indicated a permanent BCRL (Stage II-III). Looking at the time of first appearance of BCRL, we found out that almost 90% of all arm lymphedemas occurred during the first two years after radiotherapy; towards a slow but continuous increase of BCRL frequency during follow-up time thereafter [Table 2]. The questionnaire analysis showed inconsistent answers in 17 cases: in nine cases the lymphedema was pointed out clearly in LBCQ-D and we classified it as such; in eight cases the inconsistence was present in both questionnaires. Last mentioned was *e.g* negation of swelling but report of lymphedema related symptoms as subjective heaviness, tenderness and/or pain. Fortunately, in these eight cases a clear classification of symptoms could be supplemented after telephone consultation.

**Table 2:** Occurrence of arm lymphedema (BCRL)

Years after radiation	*N*° (%)
0–2	61 (87.1)
3–5	+ 1 (88.6)
6–10	+ 3 (92.9)
11–15	+ 1 (94.3)
missings	4 (5.7)

### Analysis of risk factors for the incidence of lymphedema

**Fig. 1:**
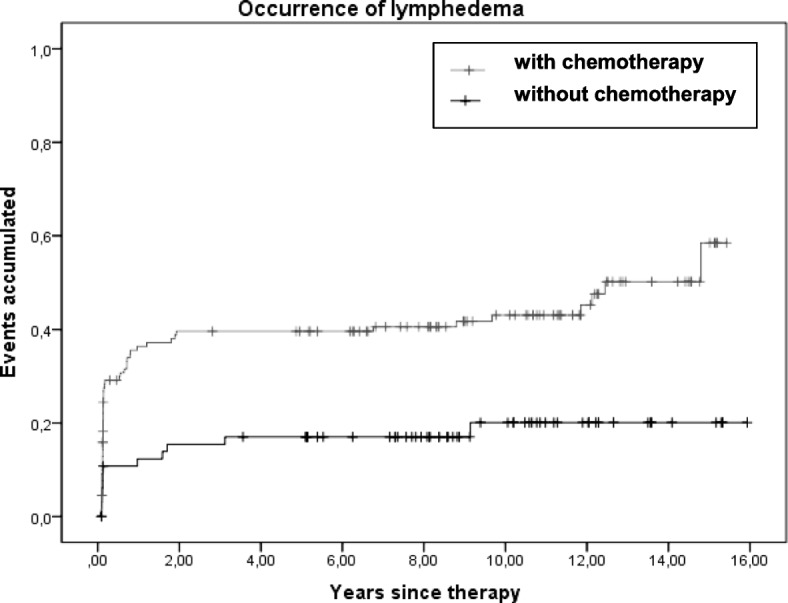
Correlation between the occurrence of lymphedema and adjuvant chemotherapy

The results of univariate risk factor analysis are summarized in Table 3. The multivariate analysis includes adjuvant chemotherapy, nodal status and postoperative complications (i.e. wound infection, hematoma and/or seroma formation) as significant parameters of univariate analysis. Statistically, only chemotherapy could be identified as a significant risk factor (*p* = 0.005). Postoperative complications showed a trend towards significance (*p* = 0.083) [Table 3; Fig. 1].

**Table 3:** Risk factors for secondary arm lymphedema (BCRL) – univariate Kaplan-Meier-Analysis and Cox-Regression-Analysis for the subgroup of BCRL

Risk factor			
Univariate Kaplan-Meier-Analysis	Category	*p*-value	95% CI
Chemotherapy adjuvant	y/n	0.001	7.7–10.2
pN Stage	N1 vs N2 + N3	0.032	4.2–8.2
Postoperative complications	y/n	0.046	0.0–11.4
Age (CP)	CI of the median	0.228	9.6–12.2
Chemotherapy neoadjuvant	y/n	0.338	8.9–14.1
pT Stage	T1 vs. T2-T4	0.621	8.9–11.8
Grading	G1 + G2 vs. G3	0.270	10.4–19.2
Tumor size (CP)	CI of the median	0.780	9.3–12.2
Resection volume (CP)	CI of the median	0.510	10.8–18.9
N° dissected lymph nodes (CP)	CI of the median	0.348	8.2–11.1
N° positive lymph nodes	1–3 vs. > 3	0.164	6.2–10.6
Hormonal therapy	y/n	0.889	9.1–11.5
Radiation SCRT	y/n	0.762	11.5–18.1
Radiation boost	y/n	0.332	9.8–19.8
Cardiovascular comorbidity	y/n	0.512	10.2–19.4
Other comorbidities	y/n	0.927	4.9–12.5
Tumor recurrence (total)	y/n	0.949	7.2–14.1
Multivariate Cox-Regression-Analysis	Patients with BCRL	*p*-value	HR; 95% CI
Chemotherapy adjuvant	58/133 (43.6%)	0.005	2.5; 0.21–0.76
Postoperative complications	5/9 (55.6%)	0.083	2.3; 0.18–1.11
pN Stage	16/35 (54.7%)	0.119	1.6; 0.32–1.10

*CI* Confidence interval, *CP*: Continuous parameter

### Lymphedema-associated symptoms

In the SDBC-D patients were asked to point out, wether they suffered from lymphedema-associated symptoms as listed in Table 4. Chi-squared tests showed a highly significant coexistence of all tested symptoms and BCRL (*p <* 0.001) [Table 4].

**Table 4:** Lymphedema-associated subjective symptoms and coincidence with secondary arm lymphedema (BCRL)

Symptom	y/n	Number of patients (%)	Concomitant with lymphedema (%)	Chi-squared test (*p*)
Chronic skin damage				< 0.001
	*Yes*	39 (19.5)	23 (59.0)	
	*No*	153 (26.5)	42 (57.5)	
Fibrosis				< 0.001
	*Yes*	67 (33.5)	34 (50.7)	
	*No*	127 (63.5)	33 (26.0)	
Pain				< 0.001
	*Yes*	77 (38.5)	43 (55.8)	
	*No*	116 (58.0)	24 (20.7)	
Peripheral neurologic symptoms				< 0.001
	*Yes*	60 (30.0)	40 (66.7)	
	*No*	132 (66.0)	28 (21.2)	
Impairment of shoulder/arm movement				< 0.001
	*Yes*	56 (28.0)	32 (57.1)	
	*No*	137 (8.5)	34 (24.8)	

### Quality of life

With the questionnaires we received a total of 186 answers with 14 answers missing. The median NRS score was 8 points (range, 0–10 points); 10 points indicating “the best possible QoL imaginable” and 0 points “the worst possible QoL imaginable”. Patients without BCRL scored a median of 8 points (range, 1–10 points) at the QoL score, while patients with BCRL scored a median of 7 points (range, 0–10 points). The Chi-squared test was used to analyse the correlation between a QoL-score < 8 points and higher rate of BCRL: Of all patients having a QoL-Score < 8; 43.2% (*n* = 54) suffered from BCRL, while only 18% (*n* = 11) of patients with a QoL-Score ≥ 8 had that condition. The high significant correlation between lower QoL-score (< 8) and higher rate of BCRL (*p* = 0.001) consistently indicates a poorer QoL in patients with lymphedema.

### Recurrence rate

15 (7.5%) of the included patients meanwhile had a history of recurrence [local recurrence n = 7 (3.5%), lymph node recurrence: n = 2 (1%), remote metastasis n = 6 (3%)]. In univariate analysis the incidence of cancer recurrence had no significant influence on the development of BCRL (p = 0.949)

## Discussion

Arm lymphedema is one of the major long-term complications after multimodal breast-conserving treatment of nodal positive breast cancer [7, 11, 12, 23, 24]. It highly impacts the patient’s QoL and can cause anxiety and psychological impairment [7, 25]. We could observe a significant correlation between BCRL and lymphedema-associated symptoms, such as pain in the affected arm, skin fibrosis and impaired shoulder/arm movement altogether. Currently, some evidence exists suggesting that the negative impact of BCRL on patients is not necessarily related to the grade of limb volume increase [7]. Even when objective measurements are the gold standard in the detection of BCRL, they might not always correspond to the impact it has on the patients subjective QoL [11]. Therefore, the role of subjective, symptom-based measurement should be emphasized. This was the primary reason for the development of the specific questionnaire LBCQ. Armer *at al* could demonstrate its validity of predicting BCRL solely by analysing self-reported symptoms [9].

In this study, we focused on breast cancer patients with nodal positive status and standard treatment. The analysed treatment decade, the beginning of the XXI^st^ century, preceded the implementation of personalized breast cancer treatment [26, 27]. Back then, all patients received a complete axillary lymph node dissection (ALND). An additional irradiation of supraclavicular lymph nodes (SCRT) was only performed in high-risk patients, e.g. when more than three positive axillary nodes had been found after ALND. Nevertheless, our data has the advantage of representing a long follow up time of median 10.1 years and ranges up to 15,9 years.

The number of participants included in this study (*n* = 200) is similar to other studies published in this area, (120 < *n* < 270) [11, 12, 24, 27–29]. But there is no consistent data on the incidence of BCRL in literature. In a meta-analysis, DiSipio et al [14] analysed 72 studies and found a pooled estimated BCRL incidence of 16.6%, with numbers ranging from 8.4% up to 21.4% (including both studies that used objective measurements but also studies that used only subjective parameters of BCRL). Ozcinar et al [29] indicated a BCRL rate after ALND of 18% (objective measurement of BCRL through physicians*;* median follow-up of 64 months*)*, while Bevilacqua et al [16] reported a 5 year cumulative BCRL rate of 30.3% (objective measurement of BCRL; median follow-up of 41 months) and Armer et al [28] a BCRL rate of 35 and 43% after 24 months and 60 months, respectively (subjective measurement of BCRL).

In our symptom-reports to determinate the BCRL incidence rate, recurrent and reversible lymphedemas which would have potentially disappeared at some point on the evaluation by a physician were also included in the analysis. So we found a max. Overall BCRL rate of 35% in this survey after a median follow-up time of 10.1 years. The prevalence of lymphedema in our survey decreases markedly, when only patients with permanent BCRL are taken into account (i.e., stage II-III): these severe lymphedemas occurred in 23.5% of the cases. Fu et al. [11] showed that all their patients being objectively diagnosed with BCRL also subjectively indicated the symptom of arm swelling and more that 70% reported arm heaviness. These symptoms were found to be significant for the detection of BCRL in their study [11]. This matches the symptoms we used as criteria for detecting the incidence rate of BCRL (swelling without/with pitting and arm heaviness). Furthermore, Fu et al. [11] showed that symptom-based reports are also valid instruments for detecting latent stages of BCRL. Objective assessments, such as limb volume change measurement, might possibly not detect all grades of BCRL, so it is important to have an early screening tool [9, 11, 12, 24]. In this context, we point out that several interventions, such as lifestyle consulting intervention, physical and psychological therapy, improve breast cancer related symptoms and have a positive effect on health-related QoL [30]. Therefore, a symptom-based report can be an effective instrument to detect patients with a need for such interventions, even if symptoms are at an early stage.

In our study almost 90% of BCRL occurred during the first two years after therapy. These findings confirm current data, in which the incidence seems to increase over time, at least up to 24 months after breast cancer diagnosis or surgery. Nevertheless, we also observed that some cases continue to accumulate beyond this period of follow up, but at a slower pace. This was also reported by some studies [14, 31].

We could only find a small number of significant risk factors for the occurrence of BCRL: These were adjuvant chemotherapy, postoperative complications and nodal stage in univariate analysis and adjuvant chemotherapy in multivariate analysis [HR = 2.5], whereas postoperative complications showed a trend towards significance in multivariate analysis [HR = 2.3]. These results are in conformity with other studies describing axillary dissection to be one of the most important risk factors of BCRL [14, 17, 32–35]. This points out the importance of early diagnosis and sentinel lymph node procedures in the prevention of this devastating disease, as other independent risk factors found in our study seemed to have little involvement on the onset of the condition. A clear association between chemotherapy and an increased risk of BCRL has already been described in some studies but the pathogenesis of this phenomenon remains unclear [14, 34–36]. Cormier et al [12] found a significant association between postoperative complications (particularly after axilla surgery) and increasing limb volume change. We found a nearly significant correlation between postoperative complications and lymphedema. Nevertheless, these findings should be interpreted with caution due to the small number of postoperative complications in our survey (*n* = 9).

The negative effect of additional axillary radiation described in the literature [10, 29, 36, 37] could not be tested in our study, as we performed no “real”/full-dose axillary irradiation.

The median value of 8 out of 10 points in the NRS for the (global) QoL was rather high, and we could show a high correlation between lower QoL and the occurrence of BCRL. Radiotherapy itself does not seem to cause low QoL. In other studies, e.g. in long-term cervical cancer survivors submitted to radiotherapy, global QoL was impaired, but improved during follow-up and eventually reaching levels comparable to that of the reference (normal) population [38]. Therefore, the lower QoL in our study might be due most likely to lymphedema. It would be interesting to analyse in further studies if this lower QoL is permanent, lymphedema being a lifelong chronic condition.

The limitations of our study were due to the retrospective analysis and to the subjective report of symptoms. This first aspect generated incomplete information e.g. on time to recurrence and causes of death. Also, the dependence on subjective information without additional clinical examination might have generated so called “recall bias” with underestimation of lymphedema within a mostly elderly population due to memory lapses or poor capacity of self-inspection. Using a second questionnaire (SDBC-D) to assess the presence of subjective symptoms contributed to the reduction of single questionnaire biases and resulted in a better evaluability of given answers.

The inclusion of recurrent swelling episodes, on the contrary, might have led to an overestimation of the BCRL rate. Patient inclusion based on replying to the questionnaire could also have caused biases. Symptom-free patients might easily refuse participation in symptom recollection, whereas affected subjects find more meaning in filling up a form.

In spite of these limitations, our study design allowed a very long follow up, retrieving important information on the long-term incidence of this associated condition. Although the global quality of life at the date of survey was measured exclusively with a Numerical Rating Scale, not allowing a selective differentiation of causative factors, it clearly indicated a chronic impairment of QoL in these patients.

## Conclusion

In summary, nearly 90% of the BCRL occurred during the first two years after radiation. Adjuvant chemotherapy and postoperative complications were the main risk factors in promoting BCRL. We recommend a symptom-based approach for detecting even latent stages of BCRL allowing targeted interventions to improve breast cancer related symptoms (including the arm lymphedema itself) and also the health-related QoL.

## Abbreviations

ALND: Axillary Lymph Node Dissection
BCRL: Breast Cancer Related Lymphedema
CI: Confidence Interval
CP: Continuous parameter
DEGRO: German Society of Radiation Oncology
HER2: Human Epidermal growth factor Receptor 2
LBCQ: Lymphedema Breast Cancer Questionnaire
LBCQ-D: Lymphedema Breast Cancer Questionnaire (in German)
NRS: Numerical Rating Scale
QoL: Quality of Life
SCRT: Supraclavicular Radiation Therapy
SDBC-D: Structure Demand for Breast Cancer Questionnaire (in German)
SEER: The Surveillance, Epidemiology, and End Results Program of the National Cancer Institute
SPSS: Statistical software package
TNM: Tumor/Node/Metastasis – classification system
WBI: Whole-Breast Irradiation
